# Fungal Diversity Associated with Hawaiian *Drosophila* Host Plants

**DOI:** 10.1371/journal.pone.0040550

**Published:** 2012-07-20

**Authors:** Brian S. Ort, Roxanne M. Bantay, Norma A. Pantoja, Patrick M. O’Grady

**Affiliations:** Department of Environmental Science, Policy and Management, University of California, Berkeley, California, United States of America; University of Minnesota, United States of America

## Abstract

Hawaiian *Drosophila* depend primarily, sometimes exclusively, on specific host plants for oviposition and larval development, and most specialize further on a particular decomposing part of that plant. Differences in fungal community between host plants and substrate types may establish the basis for host specificity in Hawaiian *Drosophila*. Fungi mediate decomposition, releasing plant micronutrients and volatiles that can indicate high quality substrates and serve as cues to stimulate oviposition. This study addresses major gaps in our knowledge by providing the first culture-free, DNA-based survey of fungal diversity associated with four ecologically important tree genera in the Hawaiian Islands. Three genera, *Cheirodendron*, *Clermontia*, and *Pisonia*, are important host plants for *Drosophila*. The fourth, *Acacia*, is not an important drosophilid host but is a dominant forest tree. We sampled fresh and rotting leaves from all four taxa, plus rotting stems from *Clermontia* and *Pisonia*. Based on sequences from the D1/D2 domain of the 26S rDNA gene, we identified by BLAST search representatives from 113 genera in 13 fungal classes. A total of 160 operational taxonomic units, defined on the basis of ≥97% genetic similarity, were identified in these samples, but sampling curves show this is an underestimate of the total fungal diversity present on these substrates. Shannon diversity indices ranged from 2.0 to 3.5 among the Hawaiian samples, a slight reduction compared to continental surveys. We detected very little sharing of fungal taxa among the substrates, and tests of community composition confirmed that the structure of the fungal community differed significantly among the substrates and host plants. Based on these results, we hypothesize that fungal community structure plays a central role in the establishment of host preference in the Hawaiian *Drosophila* radiation.

## Introduction

Insect groups have developed co-evolutionary relationships with plants since the origin of both lineages [1]–[5]. Likewise, insects and microbes have evolved close relationships, often linked to nutrition or reproduction [6]–[10]. A different type of relationship is seen in saprophagous insects, which require microbes to help them break down plant material and make nutrients available for uptake. Such a system might not necessarily have as tight a co-evolutionary relationship as some traditional examples of plant-insect interactions, but will nonetheless display signatures of both long- and short-term symbioses present within this community.

*Drosophila* species rely on fungi, especially yeasts, and bacteria to break down plant material and provide suitable substrate and nutrition for developing larvae. The three-way interaction between *Drosophila*, yeasts and cacti has served as a major ecological and evolutionary model system [11]–[14]. When cacti are damaged or begin to senesce, bacteria and fungi initiate breakdown of plant tissues. Decomposition releases volatile compounds that attract one or more *Drosophila* species as they move throughout the environment searching for suitable feeding and oviposition substrates. *Drosophila* inoculate necrotic tissue with fungi carried on their bodies or via defecation or regurgitation. The fungi grow vigorously, further breaking down plant tissues, releasing essential nutrients for larval development, and producing additional volatile compounds that attract more *Drosophila* to the rot [15], [16]. Developing larvae bore through the necrotic tissue, opening new areas for fungi to colonize. Over 100 species of *Drosophila* have evolved associations between cacti and fungi in New World deserts over the past ∼20 million years [17]–[22], capitalizing on this diverse plant resource.

The endemic Hawaiian Drosophilidae, a clade composed of 559 described and several hundred known but undescribed species [23], is one of the best known examples of an adaptive radiation in nature. The group is strongly supported as monophyletic [24]–[31]. Hawaiian *Drosophila* display extensive ecological diversity, utilizing ∼40% of the angiosperm families native to the Hawaiian Archipelago. Magnacca *et al.* [32] reviewed the rearing records of Hawaiian Drosophilidae, listing over 1100 individual records from 326 species (∼59% of the described species in this group). These data show that Hawaiian *Drosophila* often depend on a particular host plant taxon for oviposition and larval development. Furthermore, most Hawaiian *Drosophila* are specialists on a particular part of the plant (leaves, bark, fruits, sap flux). This fine-scale niche partitioning may, in part, explain the impressive morphological, taxonomic and genetic diversity of Hawaiian Drosophilidae [33]–[35]. These rearing records, coupled with a comprehensive phylogenetic analysis of the Hawaiian *Drosophila* [28], yields a picture of host plant use throughout the evolutionary history of this group.

**Figure 1:**
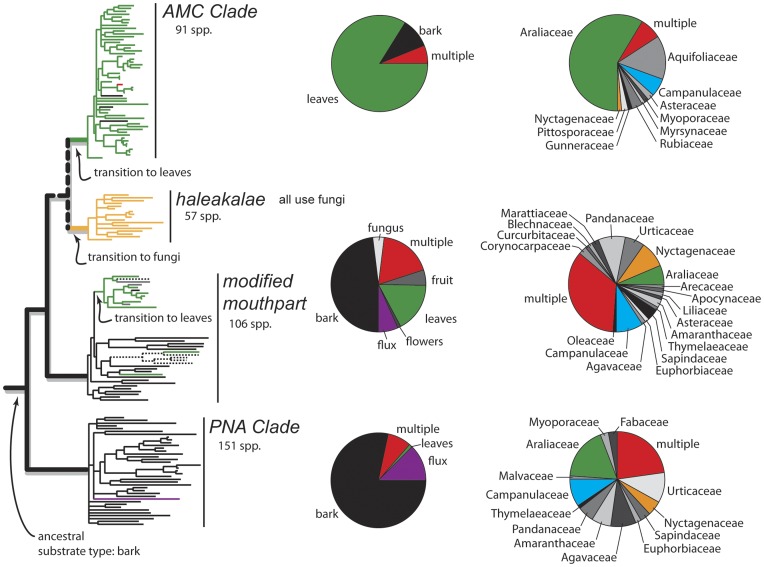
Summary of substrate and host plant use in Hawaiian *Drosophila*. Phylogenetic relationships of major lineages in Hawaiian *Drosophila* are shown with substrate type mapped onto the tree (after [28]). Rearing record data (after [32], updated with [77]) are shown in the pie charts. The first column depicts substrate types for each of the main clades (AMC, PNA and modified mouthpart), the second column shows host plant family. While these analyses are not entirely comparable because not all taxa in the phylogeny have rearing data and not all reared species were sampled for the phylogeny, they do show the same trends. First, there is a major shift from bark and stems (black) in the basal lineages to leaves (green) in the AMC and part of the modified mouthparts clades. Taxa using multiple substrate types (e.g., stems and leaves) are shown in red. Second, there is much greater diversity in host plant family usage in the picture wing and modified mouthparts clades owing to the radiation on Araliaceae within the AMC clade. The three host plant families examined in this study are color-coded on the pie charts are follows: Araliaceae (*Cheirodendron*, green), Campanulaceae (*Clermontia*, blue), and Nyctagenaceae (*Pisonia*, orange). Taxa using multiple host plant families are shown in red.

The ancestral substrate type in Hawaiian *Drosophila* is hypothesized to have been bark [28] and this is the most abundant substrate type used by the basal picture wing and modified mouthpart species groups (Figure 1). Several shifts from one type of substrate to another are evident and coincide with the origin of major lineages. For example, the transition from bark to macrofungi took place at the base of the *haleakalae* species group (Figure 1). Likewise, transitions to rotting leaves occurred several times, most notably at the base of the large *antopocerus-modified tarsus-ciliated tarsus* (AMC) clade and in the unrelated *mimica* subgroup of the modified mouthpart species group (Figure 1). Transitions from one type of substrate to another also occur within major lineages (Figure 1). For example, multiple transitions back to bark from a leaf-breeding ancestor have occurred in the modified mouthpart species group and AMC clade. Furthermore, independent transitions to fruits, macrofungi and sap fluxes are seen in the modified mouthpart and picture wing groups (Figure 1).

There is currently little information addressing how the fungal component of larval diet might be altered as Hawaiian *Drosophila* species switch from one plant host to another. The wealth of information on the cactophilic *Drosophila* system’s strong affiliations between the flies, the host plants, and the microbiotic community [15] provides a useful model for elucidating interactions between the endemic Hawaiian *Drosophila,* microbes, and host plants. The Hawaiian system is much more complex in terms of habitat diversity, species numbers, and numbers of interactions, but several scenarios can be proposed and tested once surveys of these taxa are made. The simplest scenario is that larval diet is primarily composed of a single, generalist fungus, found on all host plants (Table 1). Other fungal taxa may or may not be present in the system but would not have significant impacts on larval development. Such a scenario is seen in the cactophilic *Drosophila repleta* species group where the genus *Pichia* is widely used in the clade [13]. *Pichia* has also been recorded from some Hawaiian systems [36] and may play a similar role in this archipelago. An alternate hypothesis is that a small, core set of generalist fungi may serve the nutritional needs of the larvae, but that not all are required for larval viability in all substrates as long as a certain threshold number of core taxa are present (Table 1). Other fungal taxa within the system would not be tightly associated or play a role in larval development. This “threshold” scenario differs from the simple “one fungus” scenario by allowing for a more diverse fungal flora and for combinations of *Drosophila* symbionts. A third, and more complicated situation, would involve non-overlapping sets of fungi adapted to each host plant, some of which play a nutritional role for *Drosophila*, some of which do not (Table 1). This last scenario would require a high degree of discrimination by females when selecting a given substrate for oviposition and a correspondingly high variance in substrate quality with respect to larval nutrition.

**Table 1:** Hypotheses for the distribution of fungi that are nutritionally important to *Drosophila* larvae.

Hypothesis	Fungal Type (#)	Overlap Between Substrates	Drosophila Olfactory Specificity
One Fungus	Generalist (∼1)	High	Low
Threshold	Generalist (Few)	Moderate	Low
Complex	Specialist (Many)	Low (high substrate fidelity)	High

The amount of overlap in fungal community composition among plant host substrates would be expected to vary under the different hypotheses described in detail in the main text, as would the precision with which adult flies would need to be able to detect specific substrates for oviposition. Fungi not important to larval development are not considered here, although they may constitute a large proportion of the fungal diversity present on a given substrate.

Differences in fungal community between host plants and substrate types may provide the basis for host specificity in Hawaiian *Drosophila* by decomposing plant micronutrients and releasing different sets of volatile chemicals that are cues for high quality substrates [37]–[39]. Fungi ingested by larvae and adults are important in viability and growth [40]–[46], and *Drosophila* species may actively vector these between substrates through regurgitation or defecation [36], [47]–[49], while other fungi may be vectored on the exterior of the fly, yet not directly ingested. Chandler *et al.* [50] recently reported on the bacterial microbiome within several species of wild and lab-reared *Drosophila*. They found differences between the bacterial community in the lab environment and the gut of both adult and larval wild flies. Their results highlight the importance of sampling from the natural environment in which the flies are found in order to characterize the evolutionary relationships between flies, microbes, and the plants both use.

In this study, we provide a DNA sequence-based inventory of fungi associated with four ecologically important Hawaiian tree genera. We examined three genera, *Cheirodendron* (Araliaceae), *Clermontia* (Campanulaceae), and *Pisonia* (Nyctaginaceae), that serve as host plants for at least 71, 57, and 37 species of drosophilids, respectively, and another genus, *Acacia* (Fabaceae), that is a dominant tree in Hawaiian forests but is not extensively used as a host by the flies [32]. We sampled fresh and rotting leaves from all four genera, and examined additional rotting stem samples from *Clermontia* and *Pisonia*, as these are also important host substrates. The results show that fungal community structure differs significantly among the host and non-host plant genera, as well as between substrates that serve as host material for different species of drosophilids. In fact, there is very little overlap in fungal community composition among any of the plant-substrate combinations examined.

## Methods

### Substrate Sampling

All necessary permits were obtained from Hawaii Volcanoes National Park, the State of Hawaii, and the East Maui Irrigation Company for the described field studies. We collected a variety of living and rotting substrates from four genera of native Hawaiian trees on Maui (Makawao Forest Reserve) and the Big Island of Hawaii (Kohala Forest Reserve; Kipuka Puaulu & Olàa Tract, Hawaii Volcanoes National Park) in order to sample the fungal diversity present within Hawaiian forests, with a focus on those taxa important to larval development in the endemic Drosophilidae. Living and rotting leaves and rotting stems were collected from *Cheirodendron trigynum* and *Clermontia arborescens*, two of the major host plant genera of Hawaiian *Drosophila* [32]. Living and rotting leaves and rotting stems were also collected from *Pisonia umbellifera*, a minor but significant Hawaiian *Drosophila* host plant. Both living and decomposing leaves were also sampled from *Acacia koa*, a common native tree species but not a major host of Drosophilidae in the Hawaiian Islands. Sampling information is summarized in Table 2. Collections were preserved in 95% ethanol and transported to the laboratory for further analyses.

**Table 2:** Summary of sampling data and diversity indices.

Plant genus	Substrate	Code	N	Q	S	OTU	HNP	Chao1
All	All		27	786	113	159	4.68	291
Cheirodendron	Fresh leaf	CheiFrLf	5	58	26	27	3.45	48
	Rotting leaf	CheiRtLf	6	230	31	33	2.84	55
Clermontia	Fresh leaf	ClerFrLf	1	90	21	28	3.27	38
	Rotting leaf	ClerRtLf	1	91	25	37	3.37	53
	Rotting stem	ClerRtSt	2	97	15	30	3.67	55
Acacia	Fresh leaf	KoaFrLf	1	37	10	9	2.09	13
	Rotting leaf	KoaRtLf	1	88	28	32	3.24	48
Pisonia	Fresh leaf	PisoFrLf	5	25	11	15	3.19	25
	Rotting leaf	PisoRtLf	5	41	17	20	3.12	20
	Rotting stem	PisoRtLf	1	29	5	6	2.39	8

*N*, number of environmental samples;

*Q*, number of clones sequenced;

*S*, taxonomic richness; OTU, number of OTUs, 97% genetic similarity;

*H*_NP_, nonparametric Shannon diversity index; Chao1, Chao’s nonparametric estimate of species diversity.

### Molecular Methods

Microbial DNA was extracted from plant tissues using a DNeasy blood and tissue extraction kit (Qiagen) following the manufacturer’s protocol. We used PCR primers NL1 and NL4 [51] to amplify the D1/D2 domain of the 26S rDNA gene. Thermocycling conditions, modified after Kurtzman and Robnett [51], included an initial denaturation step of 95°C for 10 minutes, followed by 30 cycles of denaturation at 95°C for 30 seconds, annealing at 52–54°C for 30 seconds, and extension at 72°C for 1 minute. A final extension of 5 minutes at 72°C completed the reaction. PCR products were cloned into pCR2.1 using the TOPO TA Cloning kit and transformed into TOP10 chemically competent cells (Invitrogen) following the manufacturer’s instructions. We reamplified the D1/D2 domain from the cloning vector using T7 promoter and M13 reverse primers (Invitrogen) under the same reaction conditions as above except using a 5-minute initial denaturation at 95°C and an annealing temperature of 55°C. PCR products were cleaned using a exonuclease I/shrimp alkaline phosphatase protocol and sequenced in both directions using the NL1 and NL4 primers at the UC Berkeley Sequencing Facility. All new fungal sequences have been deposited in GenBank (accession numbers JX241700– JX242452).

### Diversity Analyses

Sequences were assembled and edited in Geneious (Biomatters Ltd), trimming terminal regions with >5% probability of error per base call. We tested for chimeric sequences using the ChimeraSlayer module [52] in *mothur* [53] with the dataset serving as its own reference for identifying potential pairs of chimeric “parents.” Each rDNA sequence was queried against GenBank using BLAST [54] and assigned to the BLAST hit with the highest bit score. In the case of multiple GenBank sequences with equal bit scores, one hit was arbitrarily chosen to represent the query sequence and all subsequent sequences that gave the same BLAST results. Results were binned based on the following approximations: if the sequence similarity between the BLAST hit and the query was 95% or greater, the rDNA sequence was considered to belong to the same genus as the top hit; if less than 95% similar, the query was considered to be “near” the genus of the top hit. We did not attempt to identify sequences to the level of species.

Assignment of sequences to operational taxonomic units (OTU) and subsequent genetic diversity calculations were carried out in *mothur* [53]. We aligned the sequences with and without the GenBank sequences using the E-INS-i method in MAFFT [55] on the CIPRES gateway [56]. Duplicate sequences were removed, a genetic distance matrix was constructed in PHYLIP v3.69 [57], and run through the hcluster module of *mothur* to determine each sequence’s OTU affinity using the furthest neighbor calculation. Values of over 1% genetic divergence at the ascomycetous D1/D2 domain of 26S rDNA have been proposed as representing differentiation at the species level [51], [58], so we defined OTUs rather conservatively as containing individuals with ≥97% genetic similarity. We used the *Venn* module in *mothur* to calculate the number of shared OTUs among the different fungal communities. For each collection (*e.g.*, CheiFrLf, CheiRtLf, ClerFrLf), we calculated rarefaction curves and the Chao-1 index [59] to evaluate our sampling effort. The parametric (*H*) and nonparametric (*H*_NP_) Shannon-Weiner diversity indices were used to compare diversity. *H*_NP_ is more appropriate for comparisons within our study because of the likelihood of a non-negligible number of undetected species [60]. However, because most other published studies have relied on the parametric index *H* to calculate diversity, we also calculated it for the sake of comparison, with the understanding that it will be biased downward [60]. We tested for differences in community structure across the various substrates using homogeneity of molecular variance (HOMOVA) [61], analysis of molecular variance (AMOVA) [62], and unweighted UniFrac [63], correcting for multiple comparisons using Q-Value [64]. For the three types of analyses, an initial test for significance over all data was performed. If this test was significant, all possible pairwise comparisons (45 possible) between plant-substrate combinations were performed (*e.g.,* CheiFrLf *vs*. CheiRtLf, CheiFrLf *vs*. PisoFrLf, *etc.*). AMOVA tests for differences between the diversity present in each community and the diversity of all the communities pooled. Significant results indicate that genetic composition differs among communities, but that they may or may not differ in amount of genetic diversity. HOMOVA tests for differences in the amount of genetic diversity in each community. UniFrac tests for shifts or pivots in the genetic structure of populations wherein the amount of diversity may be the same but the composition differs among communities. If both the AMOVA and HOMOVA tests are significant, UniFrac offers little additional information [65].

### Phylogenetic Analyses

An unrooted neighbor-joining (NJ) tree derived from a second MAFFT alignment, including our sequences and the best hit GenBank sequences, was constructed to help visualize taxonomic delimitations. We used PAUP* [66] using the GTR model of evolution with 1000 bootstrap replications on the 50% majority-rule consensus tree. We then mapped the host plant and substrate type of each sample and the class and species identity of the top BLAST hits onto the NJ tree using MacClade [67].

## Results

**Figure 2:**
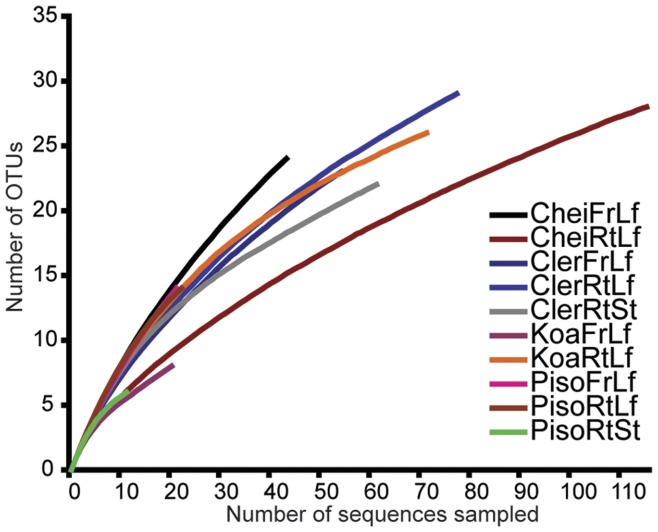
Rarefaction analyses of observed fungal OTU richness within ten plant/substrate sample types. OTUs were defined at the 97% genetic similarity cut-off using the furthest-neighbor clustering method in *mothur*.

A remarkable breadth of fungal diversity was present in tissues from four Hawaiian tree species. We sequenced a total of 1460 clones, representing 27 different plant samples from 10 different substrates (Table 2). Of these, 786 were identified by BLAST as fungal sequences, while the rest included a combination of host plant cpDNA, protozoa, or bacterial sequences and were removed from the data set. No chimeric sequences were detected. Of the fungal sequences, 529 were unique, representing 13 fungal classes and 118 genera or “near genera” based on BLAST similarity. The sequences grouped into 160 OTUs using a ≥97% similarity cutoff in *mothur*, a 36% increase over the number of genera or near genera we identified in our BLAST searches. For comparison, using a ≥95% genetic similarity cutoff to match the similarity cut-off used in our BLAST assignments yielded 133 OTUs, a 13% increase over the 118 BLAST identifications. The number of OTUs discovered was strongly dependent on sequencing effort per sample, and ranged from six OTUs identified from 29 sequenced clones for *Pisonia* rotting stems, to 37 OTUs from 91 clones for *Clermontia* rotting leaves (Table 2). Rarefaction curves began to asymptote for those samples with approximately 90 or more sequences (CheiRtLf, ClerFrLf, ClerRtLf, ClerRtSt, KoaRtLf), but no sample in this study actually reached the asymptotic phase (Figure 2).

**Figure 3:**
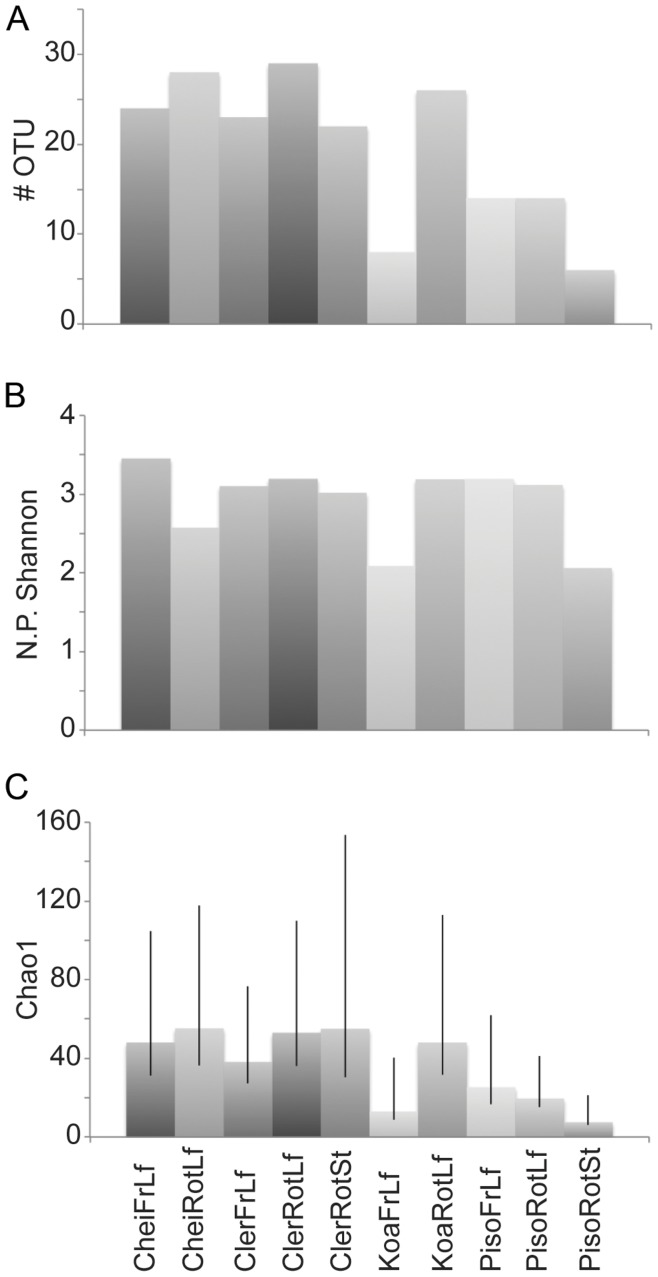
Diversity summary statistics for fungal communities. Diversity is described for ten plant/substrate types, based on an OTU definition of 97% genetic similarity. (a) The number of OTUs identified in the sample. (b) The non-parametric Shannon diversity index, *H*_NP_. (c) Chao1 diversity.

The estimate of the parametric Shannon diversity index *H* for all samples combined in our study was 4.42, while the non-parametric equivalent *H*_NP_ was 4.68. For individual samples, *H* ranged from 1.7 in *Pisonia* rotting stems (6 OTU/29 sequences) and *Acacia* fresh leaves (9/37) to 2.9 in *Acacia* rotting leaves (32/88) and *Cheirodendron* fresh leaves (27/58), while *H*_NP_ ranged from 2.1 (*Pisonia* rotting stems and *Acacia* fresh leaves) to 3.5 (*Cheirodendron* fresh leaves) (Figure 3). Diversity (*H_NP_*) was lowest in *Pisonia* rotting stems, while *Cheirodendron* fresh leaves had the highest diversity, but with several other samples close behind. There were no clear patterns of differences in diversity between all of the fresh versus all of the rotting substrates, nor between all of the substrates from one host plant compared to those from any other host plant.

**Figure 4:**
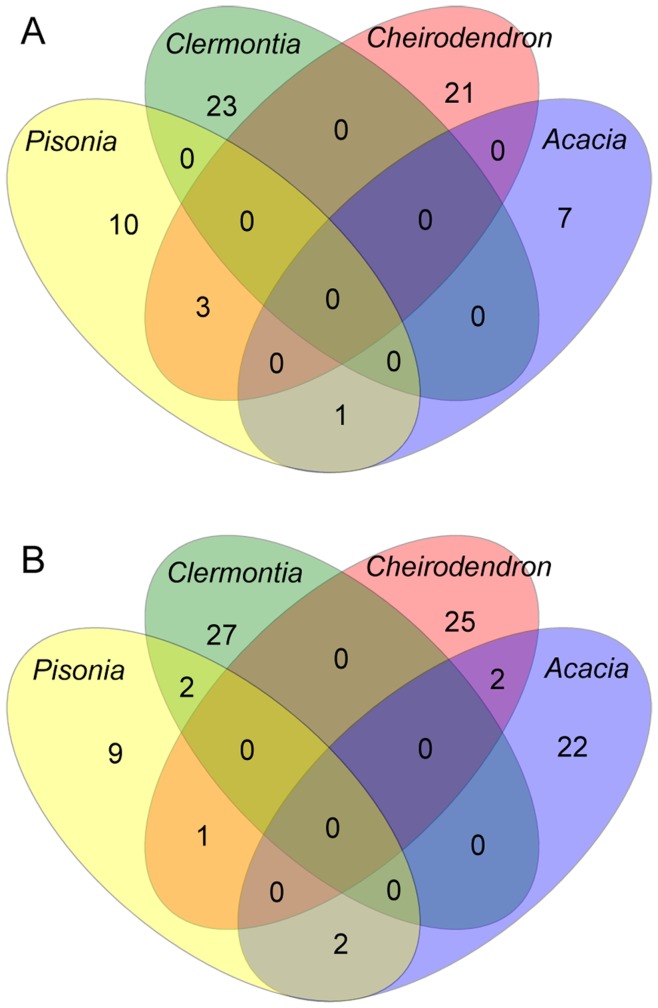
Overlap in fungal community composition on host plants. Venn diagrams display the degree of overlap of OTU identity among (a) fresh leaves from four Hawaiian tree genera, and (b) rotting leaves from the same genera. Other permutations of four-way comparisons are not shown but were qualitatively similar, *i.e.*, very little sharing of OTUs among plants and substrates. OTUs were defined using a 97% genetic similarity cut-off.

**Figure 5:**
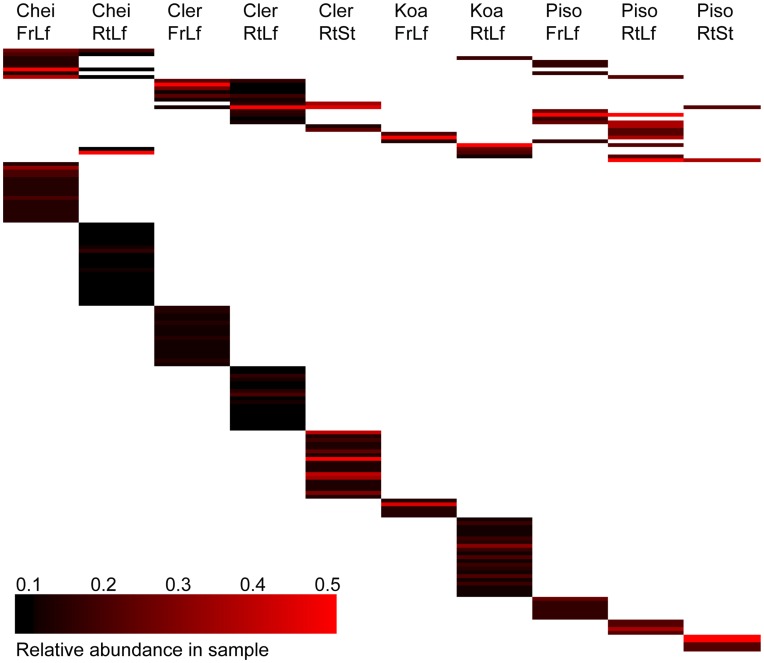
Heat map plotting composition and distribution of fungal OTUs among plant/substrate types. Each row represents a single OTU, the color of the bar in each column indicates the relative abundance of that OTU within each sample type. Red indicates greater abundance, black indicates lower abundance, and white indicates the absence of the OTU in that sample. The deficiency of shared OTUs results in the stairstep pattern seen in the lower portion of the figure.

Understanding similarities and differences in community composition between samples in our study could potentially allow us to identify key microbial taxa in the Hawaiian *Drosophila*-host plant system. Venn diagrams were used to plot the degree to which OTUs were shared between the samples. Figure 4 illustrates that major differences in community composition exist among the plant and substrates sampled. Similarly, a heat map illustration demonstrates that both OTU identity and abundance differ among the sample types (Figure 5). Indeed, both homogeneity of molecular variance (HOMOVA) and analysis of molecular variance (AMOVA) tests over the entire data set showed that fungal community structure was significantly different among all the samples. Furthermore, in the HOMOVA, 24 of the 45 possible pairwise comparisons between samples (*e.g.*, CheiFrLf *vs*. CheiRtLf, ClerRtLf *vs*. PisoRtSt, *etc.*), were significant after correcting for multiple tests using the false discovery rate. Thirteen of the twenty-four pairwise comparisons included *Pisonia* as at least one of the compared samples, ten included *Clermontia*, and eight and six comparisons included *Acacia* or *Cheirodendron*. In the AMOVA, 39 pairwise comparisons were significant and 6 were not significant. Five of the six non-significant pairwise AMOVA results included *Pisonia*, three included *Acacia*, two included *Cheirodendron*, and one included *Clermontia*. As expected given the HOMOVA and AMOVA results, the unweighted phylogenetically-based UniFrac test revealed significant differences in community structure in the global test across all samples, as well as in all pairwise comparisons.

**Figure 6:**
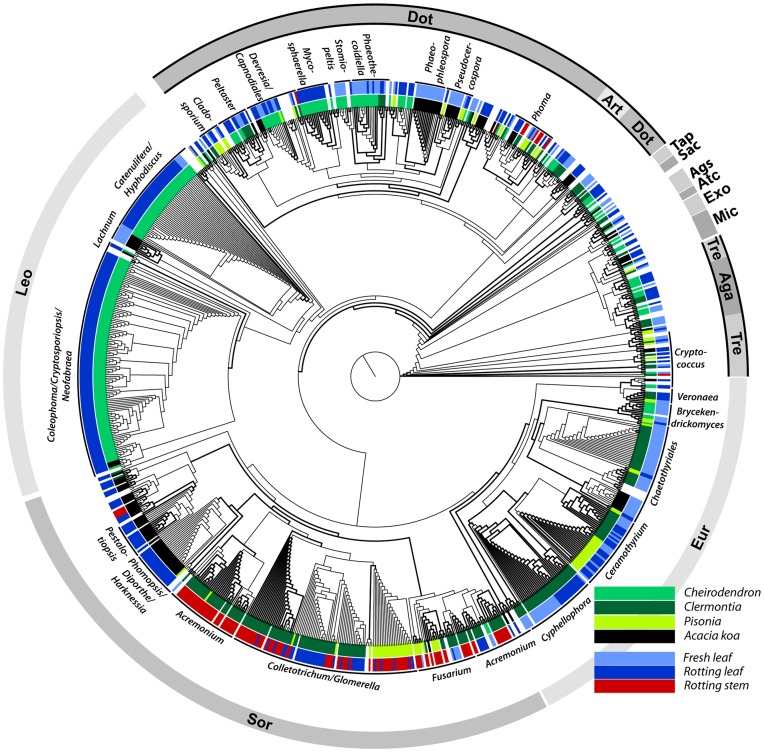
Neighbor joining phylogenetic tree. The tree is composed of fungal sequences generated in this study and their respective closest BLAST hits. Branches with bootstrap support ≥70 shown in bold. The two colored rings surrounding the tree indicate the plant genus (innermost ring) and the type of substrate (second ring) from which the fungal sequences in this study were obtained. Fungal genera are indicated outside the substrate ring. Fungal classes are indicated by the grey outermost ring. Classes are abbreviated as follows: Aga, Agaricomycetes; Ags, Agaricostilbomycetes; Art, Arthoniomycetes; Atc, Atractiellomycetes; Dot, Dothideomycetes; Eur, Eurotiomycetes; Exo, Exobasidiomycetes; Leo, Leotiomycetes; Mic, Microbotryomycetes; Sac, Saccharomycetes; Sor, Sordariomyctetes; Tap, Taphrinomycetes; Tre, Tremellomycetes. Classes that came out paraphyletic on the tree (Dot and Art; Tre and Aga) are indicated with a black border around the grey bar.

Combining all fungal sequences with their best corresponding BLAST hit, we produced a NJ tree with 956 taxa (Figure 6). We identified a broad diversity of taxa, representing 13 fungal classes and 118 genera or “near genera” among our samples, plus five different categories of uncultured or unidentified fungi. In terms of the number of clones obtained, the results are dominated by four large classes within the subphylum Ascomycota, Pezizomycotina: Dothideomycetes (162 clones), Leotiomycetes (160), Sordariomycetes (183), and Eurotiomycetes (148), and a few examples of Arthoniomycetes (3), Taphrinomycetes (2) and Saccharomycetes (2). Several classes of Basidiomycota were also detected: Tremellomycetes (20), Agaricomycetes (14), Microbotryomycetes (6), Exobasidiomycetes (4), Atractiellomycetes (1), and Agaricostilbomycetes (4). The remaining 67 clones were either unidentified in GenBank or their GenBank classification did not match their placement on the tree. Large portions of the tree are dominated by one or another of the plant/substrate combinations. In the most striking example, Leotiomycetes were found almost exclusively in the rotting leaves of Cheirodendron. Several portions of the Sordariomycetes subtree represent fungi found primarily on rotting leaves of *Acacia* or *Clermontia* and rotting *Clermontia* stems. In contrast, sequences representing the fungal genus *Ceramothyrium*, within Eurotiomycetes, reveal a generalist fungus found in fresh and rotting leaves of *Pisonia*, *Acacia*, and *Clermontia*. In two cases, the phylogenetic tree nested one class within another. The Agaricomycetes were placed within the Tremellomycetes, and the Arthoniomycetes were placed within the Dothideomycetes. Neither placement received bootstrap support >50, however, and may be an artifact.

## Discussion

### Fungal Diversity

In spite of the large diversity of fungi discovered in our survey, our numbers are certainly underestimates, both because the 97% similarity cut-off is fairly conservative, and because rarefaction curves and Chao1 estimates indicate that our clone libraries captured a fraction of the actual fungal diversity present (Figures 2 & 3). This reflects a common issue that is both an advantage and a frustration in DNA-based surveys of microbes, *e.g.*, [68], [69]. While culture-free molecular approaches detect higher levels of diversity than techniques that rely solely on culturing, morphological descriptions and fine-scale ecological characterizations of taxa discovered are not possible.

As more data on microbes accumulate it is apparent that total alpha-level diversity still exceeds our estimates. Our results are similar to other studies utilizing Sanger sequencing of clone libraries. For example, Fierer *et al.* [68] observed 216 OTUs (using a 97% sequence similarity threshold) in 310 sequences from ten individual soil samples from a continental (Amazonian) rainforest, and comparable numbers from desert and temperate soils. O’Brien *et al.* [69] found 133 OTUs in 212 sequences from 100 1-m^2^ collections of leaf litter under temperate hardwood and pine forests. New techniques, such as high-throughput pyrosequencing, allow higher sampling rates but do not immediately solve the challenge of saturating the collection curve, as Buée *et al.* [70] discovered while surveying temperate forest soil fungi. These authors’ estimates of 600–1000 OTUs per soil sample do exceed those we report here, however they still report rarefaction curves that do not reach a plateau and Chao1 estimates of taxonomic diversity that are approximately double their observed richness.

Of the three studies mentioned above, only O’Brien *et al.* [69] report the Shannon diversity index *H* (and not *H*_NP_), which ranged from 3.6–5.6. As they report similar numbers of OTUs in fewer total sequences compared to our study, their higher values for *H* probably result from a more even distribution of taxa within their samples. This may be a reflection of the general pattern of decreased taxonomic richness and less complex community structure in island systems compared to continents [71]. This result is surprising, since fungal spores should be able to travel long distances on the wind to reach even the isolated archipelago of Hawaii.

AMOVA, HOMOVA, and UniFrac analyses showed significant differences in community structure among all of the samples. The majority of sequences obtained were from Ascomycota and these showed the most obvious patterns of host plant and substrate fidelity (Figure 6). However, both Ascomycota and Basidiomycota may play important roles in *Drosophila* diet and host preference. Interestingly, the differentiation apparent in the Venn diagrams and demonstrated by the HOMOVA and AMOVA results shows that substrate (rotting leaf, fresh leaf, rotting stem) appears to be as important as host plant identity in determining the fungal community composition. Fungal specialization on plant substrates may provide the basis for flies specializing on a particular substrate within a host plant.

### Phylogenetics

There are two main patterns we observe by examining host plant and fungal associations on the fungal phylogeny. Some taxa, such as the class Leotiomycetes, appear to be specialists that may be restricted to a single plant genus. Other fungi, like *Acremonium* and *Fusarium*, are generalists that can utilize a broad diversity of plant genera and substrate types. The presence of generalists on the NJ tree is seemingly at odds with the Venn diagrams in Figure 4 that show practically no overlap in substrate use among the genetically defined OTUs. Partly this may reflect a difference in the way we grouped sequences. The 97% genetic distance cut-off used to define OTUs was appropriate to cluster sequences at roughly the species level. However, we sometimes had BLAST hits with equal scores from multiple species in GenBank, so we used a 95% sequence similarity criterion to define the tips of the NJ tree. Repeating the construction of Venn diagrams, HOMOVA, and AMOVA analyses using a 95% distance cut-off did not result in notably greater overlap in OTU niches (results not shown). Instead, there is probably a great deal of genetic diversity within the relatively poorly characterized fungal genera that we identified. In fact, the presence of polyphyletic fungal classes on the tree illustrates the need for more focused research on fungal phylogenetics.

### Implications for *Drosophila* Evolution

O’Grady and Markow (in press) have recently classified Hawaiian *Drosophila* into four guilds (broad generalist, substrate generalist, substrate specialist or true specialist) based on the breadth of feeding and oviposition substrate types they use and the phylogenetic relationships of the taxa they specialize upon. Broad generalists are able to capitalize on multiple resources spread across feeding guilds (*e.g.*, flowers, fungi, fruits) and may be reared from diverse resources. Substrate generalists are restricted to a given feeding guild (*e.g.,* fruit) but can utilize this resource across a wide array of unrelated plant species. Generalist frugivores using many different rainforest fruits are an example of a substrate generalist. Substrate specialists have adapted to a single substrate type from a single clade of host plant. Most Hawaiian *Drosophila* are classified as substrate specialists because individual species use one substrate type from a single clade (*e.g.*, leaves of Araliaceae in the case of *Drosophila waddingtoni*). True specialists are the most narrowly defined category and can use only a single type of plant resource from a single species of host plant.

Hawaiian *Drosophila* species differ in their preference for a given host plant or plant part as an oviposition substrate. Fly larvae must have the correct microbial community present in order to develop properly, and this may be the critical factor for why a fly must correctly locate a suitable host [41], [46], [72]. The current work demonstrates that fungal communities differ as a function of host plant genus and type of substrate (Figures 5 & 6). The absence of any fungi common to the substrates examined argues against the “one fungus” and “threshold” hypotheses and lends support to the “complex” hypothesis (Table 1), although data on the nutritional roles of these fungi is currently lacking and will be necessary to determine exactly how this system has evolved. These data suggest that fungal community composition may have a strong influence on the radiation of the Hawaiian *Drosophila* and the host-specificity that is a hallmark of the group. Flies rely on olfactory and gustatory cues to locate suitable feeding and oviposition substrates [73]–[76]. These cues are a combination of volatile and other chemicals produced in concert by the decomposing host plants and the microbes. Individual *Drosophila* species must be able to discern the difference between cues from substrates that are optimal, merely suitable or inappropriate.

Further investigation will be required to determine if the cues used by adult flies to locate suitable substrate are produced by the fungi present, the plant, or a combination of both. Furthermore, we know little about the role the flies play in inoculating plant substrates with the fungi their offspring will need, whether they simply carry fungi from rot to rot on their external body surfaces, or, as in the case of cactophilic *Drosophila*, inoculate rots through regurgitation and defecation [15], [16]. It has been shown that species of *Drosophila* differ in the bacterial microbiome they harbor [50], and the same may be the case for the fungal microbiome. We have initiated investigations into the composition of the fungal community residing within the digestive tracts of Hawaiian *Drosophila*. The results presented here, combined with the rearing records collected by Magnacca *et al.* (2008), provide opportunities to design focused experiments targeting not only particular *Drosophila* species, but also the fungi predicted to be the most important for the nutrition and survival of those flies.
